# Supplementary material for: Novel Driver Strength Index highlights important cancer genes in TCGA PanCanAtlas patients
Source: PeerJ. 2022 Aug 11;10:e13860. doi: 10.7717/peerj.13860 (PMC9375969; doi:10.7717/peerj.13860)
Supplement: Supplemental Information 2 [file peerj-10-13860-s002.zip › COHORTS/genes plots/cohorts/2021_8_16_14_9_distribution_cohorts_females_CHOL.pdf]

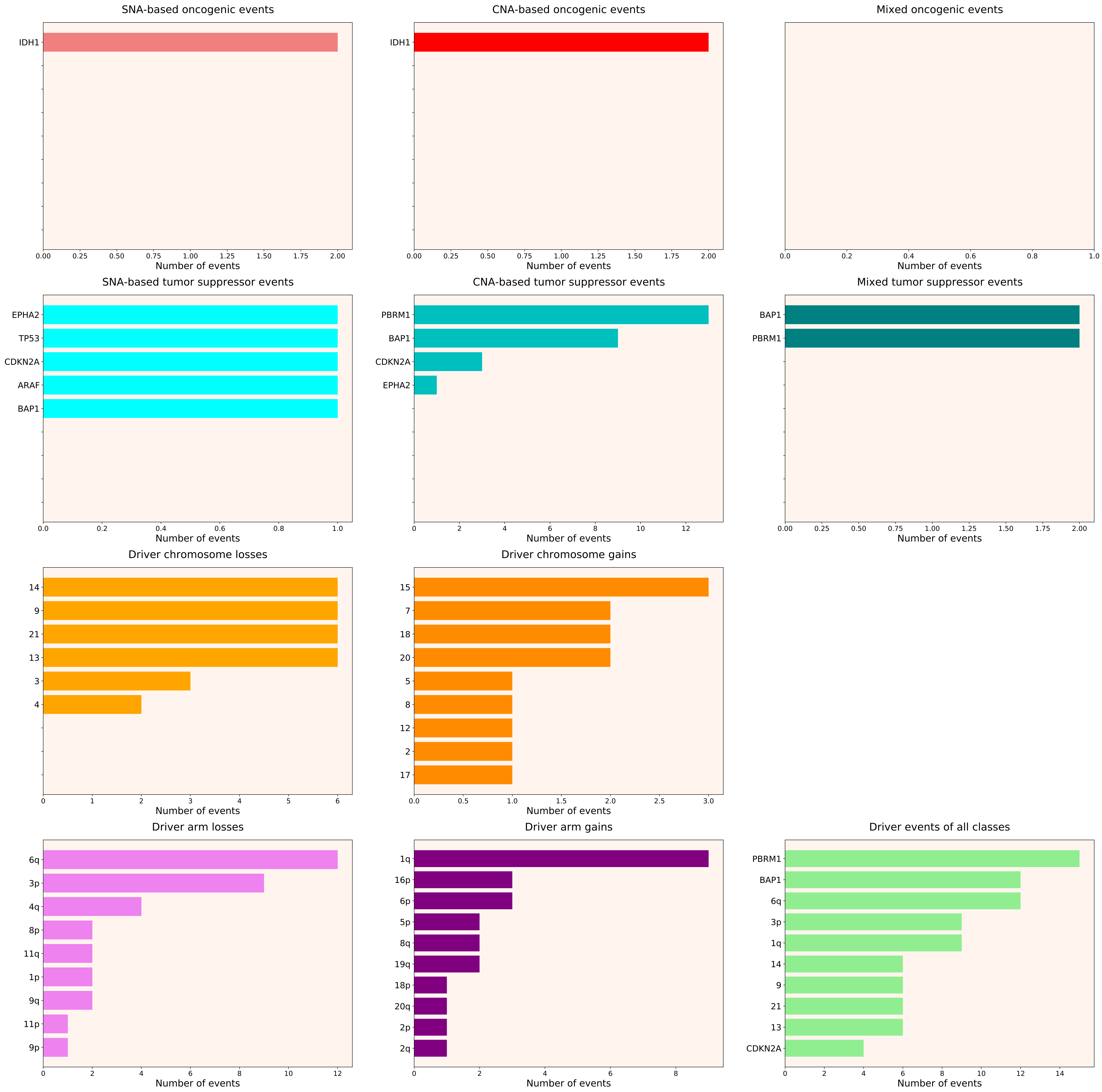

SNA-based oncogenic events
IDH1
Number of events
CNA-based oncogenic events
IDH1
Number of events
Mixed oncogenic events
Number of events
SNA-based tumor suppressor events
EPHA2
TP53
CDKN2A
ARAF
BAP1
Number of events
CNA-based tumor suppressor events
PBRM1
BAP1
CDKN2A
EPHA2
Number of events
Mixed tumor suppressor events
BAP1
PBRM1
Number of events
Driver chromosome losses
14
9
21
13
3
4
Number of events
Driver chromosome gains
15
7
18
20
5
8
12
2
17
Number of events
Driver arm losses
6q
3p
4q
8p
11q
1p
9q
11p
9p
Number of events
Driver arm gains
1q
16p
6p
5p
8q
19q
18p
20q
2p
2q
Number of events
Driver events of all classes
PBRM1
BAP1
6q
3p
1q
14
9
21
13
CDKN2A
Number of events
